# Burden of HPV related anogenital diseases in young women in Germany – an analysis of German statutory health insurance claims data from 2012 to 2017

**DOI:** 10.1186/s12879-020-05002-w

**Published:** 2020-04-22

**Authors:** Miriam Reuschenbach, Sarah Mihm, Regine Wölle, Kim Maren Schneider, Christian Jacob, Sebastian Braun, Wolfgang Greiner, Monika Hampl

**Affiliations:** 1 Department of Medical Affairs, MSD SHARP & DOHME GmbH, Haar, Germany; 2 Department of Market Access, MSD SHARP & Dohme GmbH, Haar, Germany; 3 Xcenda GmbH, Hanover, Germany; 4 Bielefeld University, Bielefeld, Germany; 5 Department of Obstetrics and Gynecology, University Hospital of Duesseldorf, Duesseldorf, Germany

**Keywords:** Human papillomavirus (HPV), Genital warts, Cervical intraepithelial neoplasia, Prevalence, Claims data, Statutory health insurance, Germany

## Abstract

**Background:**

Most individuals are infected with human papillomavirus (HPV) at least once in their lifetime. Infections with low-risk types can cause genital warts, whereas high-risk types can cause malignant tumors. The aim of this study was to determine the burden of anogenital diseases potentially related to HPV in young women based on German statutory health insurance claims data.

**Methods:**

We conducted a retrospective claims data analysis using the “Institute for Applied Health Research Berlin” (InGef) Research Database, containing claims data from approximately 4 million individuals. In the period from 2012 to 2017 all women born in1989–1992, who were continuously insured between the age of 23–25 years were identified. Using ICD-10-GM codes (verified diagnosis in the outpatient sector or primary or secondary diagnosis in the inpatient sector) the administrative prevalence (95% confidence interval) of genital warts (A63.0), anogenital diseases grade I (K62.8, N87.0, N89.0, N90.0), grade II (N87.1, N89.1, N90.1) and grade III (D01.3, D06.-, D06.0, D07.1, D07.2, N87.2, N89.2, N90.2) was calculated (women with diagnosis divided by all women).

**Results:**

From 2012 to 2017, a total of 15,358 (birth cohort 1989), 16,027 (birth cohort 1990), 14,748 (birth cohort 1991) and 14,862 (birth cohort 1992) women at the age of 23–25 were identified. A decrease of the administrative prevalence was observed in genital warts (1.30% (1.12–1.49) birth cohort 1989 vs. 0.94% (0.79–1.10) birth cohort 1992) and anogenital diseases grade III (1.09% (0.93–1.26) birth cohort 1989 vs. 0.71% (0.58–0.86) birth cohort 1992). In anogenital diseases grade III, this trend was especially observed for severe cervical dysplasia (N87.2) (0.91% (0.76–1.07) birth cohort 1989 vs. 0.60% (0.48–0.74) birth cohort 1992). In contrast, anogenital diseases grade I (1.41% (1.23–1.61) birth cohort 1989 vs. 1.31% (1.14–1.51) birth cohort 1992) and grade II (0.61% (0.49–0.75) birth cohort 1989 vs. 0.52% (0.42–0.65) birth cohort 1992) remained stable.

**Conclusions:**

A decrease of the burden of anogenital disease potentially related to HPV was observed in the younger birth cohorts. This was observed especially for genital warts and anogenital diseases grade III. Further research to investigate this trend for the upcoming years in light of varying HPV vaccination coverage for newer birth cohorts is necessary.

## Background

Human papillomavirus (HPV) infection belongs to the most frequent sexually transmitted infections in men and women worldwide [1]. Nearly all sexually active individuals will acquire at least one HPV infection in their life [2]. Although the majority of HPV infections are cleared spontaneously within a couple of months, they may become persistent with a subsequently increased risk of developing genital warts and certain cancer types [3].

HPV types capable of infecting mucosal epithelia are subdivided into low-risk and high-risk types. The low-risk types can lead to anogenital warts (condylomata acuminata). Low-risk types HPV 6 and 11 are responsible for approximately 90% of all anogenital wart cases [4]. Worldwide, several million cases of anogenital warts occur each year in both sexes, with a peak incidence between 20 and 24 years of age for women and between 25 and 29 years among men [5]. In Germany, a crude incidence rate of anogenital warts for women aged 10 to 79 years old was reported with 181 per 100,000 person years in 2010 [6].

High-risk HPV types can cause malignant conditions such as cervical intraepithelial neoplasia and cervical cancer [7]. Additionally, precancerous lesions and cancers at other anogenital sites are known to be associated with high-risk HPV. In Germany, about 4600 women are newly diagnosed with cervical cancer every year and approximately 1500 women die from cervical cancer per year [8]. It is assumed that high-risk HPV infections cause almost all cervical cancers and precancers, approximately 90% of high-grade anal, vulvar and vaginal intraepithelial neoplasias, and approximately 30, 70, and 90% of vulvar, vaginal and anal cancers, respectively [9, 10]. There are at least 12 high-risk HPV types, of which HPV 16 and 18 are responsible for 45% of cervical high-grade intraepithelial neoplasia and 70% of cervical cancers. Approximately 70–90% of HPV associated precancers and cancers at non-cervical anogenital sites are induced by HPV 16 and 18 [9].

HPV vaccination can prevent certain HPV infections and HPV-related anogenital diseases. The European Medicines Agency (EMA) authorized the first HPV vaccines in 2006 (quadrivalent vaccine against HPV 6, 11, 16 and 18) and 2007 (bivalent HPV 16 and 18) [11]. The quadrivalent vaccine may protect against HPV types causing approximately 70% of cervical cancers and 90% of genital warts [3]. Since 2016, a 9-valent vaccine is available in Germany which in addition to HPV types 6, 11, 16, and 18 also immunizes against the high-risk HPV types 31, 33, 45, 52, and 58 [12, 13]. These five additional HPV types are supposed to account for 15–20% of all cervical carcinomas [14].

In Germany, the Standing Committee on Vaccination (STIKO) at the Robert Koch Institute (RKI) is responsible for recommendations on vaccinations. These are then covered by the statutory health insurance (SHI) for all insured persons according to the recommended conditions e.g. in terms of age and gender [15]. School-based or community-based vaccination programs do not exist in Germany. In 2007, the STIKO released the first recommendation for HPV vaccination of girls in the age group of 12–17 [16]. In August 2014, the STIKO lowered the recommended vaccination age to 9–14 years. Since then, only two doses have been recommended. For catch-up vaccinations at the age of 15–17 years, the STIKO continued to recommend three doses. Since 2018, HPV vaccination is recommended for girls and boys at the age of 9–14 with catch-up until the age of 17 [17]. In the first year after the introduction of the HPV vaccination in Germany (2008), the vaccination rate was reported with 32.2% for at least one dose in the target age group of the first STIKO recommendation (12- to 17-year-old girls) [18, 19]. In 2015, the HPV vaccination rate was 31.3% in 15 year old and 44.6% in 17 year old girls (3 doses) [20]. Thus, in comparison to other countries with vaccination programs in schools, Germany has a lower immunization coverage for HPV [21]. To date, the burden of HPV related anogenital diseases has not been systematically evaluated for female birth cohorts eligible to receive the HPV vaccine after its introduction in 2007 in Germany.

The aim of this study was to assess the burden of anogenital diseases potentially related to HPV in women at the age of 23–25 based on diagnoses documented in German sickness fund data. The burden of HPV-related anogenital diseases is poorly explored in the birth cohorts who had a chance to receive HPV vaccination directly after its introduction in Germany in 2007. By assessing the first three birth cohorts (1990–1992) who were fully eligible for HPV vaccination according to the first STIKO recommendation and birth cohort 1989 which was partially eligible (this cohort turned 18 in 2007) we aimed to generate insights into the burden of potentially HPV-related anogenital diseases in these specific birth cohorts after the introduction of the HPV vaccination in Germany. HPV vaccination status of the study population could not be evaluated.

## Methods

### Data source

A retrospective claims database analysis was conducted using the “Institute for Applied Health Research Berlin” (InGef) Research Database. The database comprises anonymized healthcare claims data from approximately 8 million covered lives from about 60 different sickness funds. The data include patient demographics and characteristics, inpatient and outpatient diagnoses, surgeries and diagnostic codes, the healthcare resource utilization and costs of services for the inpatient care, outpatient care, pharmacological therapy, remedies, devices and aids, and sick leave in an anonymized case-by-case individual format. For scientific research projects, an adjusted analysis sample of the InGef database has been created which includes approximately 4 million covered lives structured to represent the German population in terms of age and gender according to the Federal Office of Statistics. The InGef sample represents about 5.5% of the German SHI population, whereas about 85% of the German population is insured by the SHI. It has been proven to have good external validity to the German population in terms of morbidity, mortality and drug use [22].

### Study population

The STIKO recommendation for HPV vaccination from 2007 included girls between 12 and 17 years. Birth cohort 1989 turned 18 in 2007 and therefore, parts of the birth cohort were too old for the HPV vaccination according to the STIKO recommendation from 2007. The female birth cohorts 1990 to 1992 were the first female birth cohorts that fulfilled the recommended age criteria for HPV vaccination according to the STIKO. Thus, this study included the first three female birth cohorts (1990, 1991, and 1992) which were fully eligible to receive HPV vaccination according to the age recommended by STIKO in 2007 and furthermore, the birth cohort 1989 which was partially eligible to receive HPV vaccination according to the STIKO recommendation. In total, the study population comprised all women in the InGef Research Database from 2012 to 2017 who were born in 1989, 1990, 1991, or 1992. All women had to be continuously observable from the age of 23 to 25, except for women who deceased. Age was determined at December 31st of each year.

### HPV related Anogenital diseases

The identification of potentially HPV-related anogenital diseases was based on International Statistical Classification of Diseases, German Modification (ICD-10-GM) codes. All potentially HPV-related anogenital diseases (cytologically or histologically derived) in the outpatient sector (verified diagnoses) and in the inpatient sector (main and secondary diagnoses) were identified (Table 1).

**Table 1:** List of ICD-10-GM codes utilized for identification of potentially HPV-related anogenital diseases

Group	Description	ICD-10-GM Code
Genital warts	Anogenital (venereal) warts (condylomata)	A63.0
Grade I	Other specified diseases of anus and rectum (AIN I & II)	K62.8
	Mild cervical dysplasia (CIN I)	N87.0
	Mild vaginal dysplasia (VAIN I)	N89.0
	Mild vulvar dysplasia (VIN I)	N90.0
Grade II	Moderate cervical dysplasia (CIN II)	N87.1
	Moderate vaginal dysplasia (VAIN II)	N89.1
	Moderate vulvar dysplasia (VIN II)	N90.1
Grade III	Carcinoma in situ of anus and anal canal (AIN III)	D01.3
	Carcinoma in situ of cervix uteri (CIN III)	D06.-
	Carcinoma in situ of endocervix	D06.0
	Carcinoma in situ of vulva (VIN III)	D07.1
	Carcinoma in situ of vagina (VAIN III)	D07.2
	Severe cervical dysplasia	N87.2
	Severe vaginal dysplasia, other	N89.2
	Severe vulvar dysplasia, other	N90.2
Carcinoma	Malignant neoplasm of cervix uteri	C53.-
	Malignant neoplasm of anus and anal canal	C21.-
	Malignant neoplasm of vulva	C51.-
	Malignant neoplasm of vagina	C52.-

### Administrative prevalence rates (APR)

The 3-year APR were calculated by dividing the total number of women in the respective birth cohort with the documentation of at least one of the defined ICD-10-GM codes by the total number of women in the respective birth cohort who were continuously observable in the observation period. The 3-year APR were reported in percent.

The 1-year APR for each age group in each female birth cohort and ICD-10-GM code group were calculated by using a similar formula: total number of women in the respective birth cohort and age group with at least one of the defined ICD-10-GM codes divided by the total number of women in the respective birth cohort and age group who were continuously observable in the respective calendar year. The 1-year APR were also reported in percent.

Furthermore, confidence intervals with 95% confidence level were calculated for the APR by applying the exact Clopper–Pearson method, which is based on the exact binomial distribution and not a large sample normal approximation and is rather suitable in case of a small n [23].

## Results

**Fig. 1:**
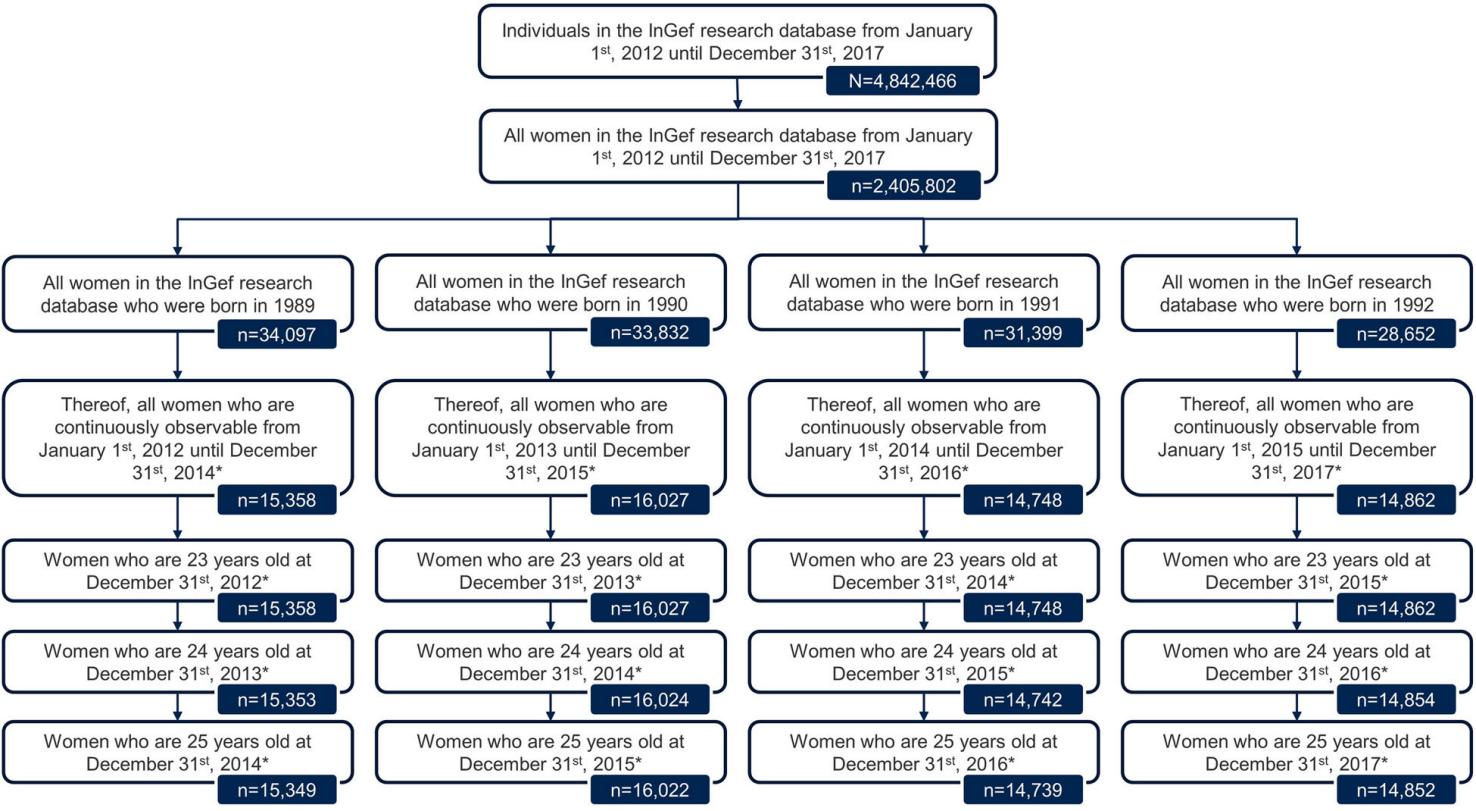
Patient flowchart. * Including patients who deceased in the respective observation period/year

The InGef Research Database included a total of 2,405,802 women from January 1st, 2012 to December 31st, 2017. In total, 15,358 (birth cohort 1989), 16,027 (birth cohort 1990), 14,748 (birth cohort 1991) and 14,862 (birth cohort 1992) women were continuously insured at the age of 23 to 25, also including women who deceased in this age period. Within the 3 years of observation a minimum of five (birth cohort 1990) and a maximum of 10 (birth cohort 1992) women deceased in the age period from 23 to 25 years (Fig. 1).

**Fig. 2:**
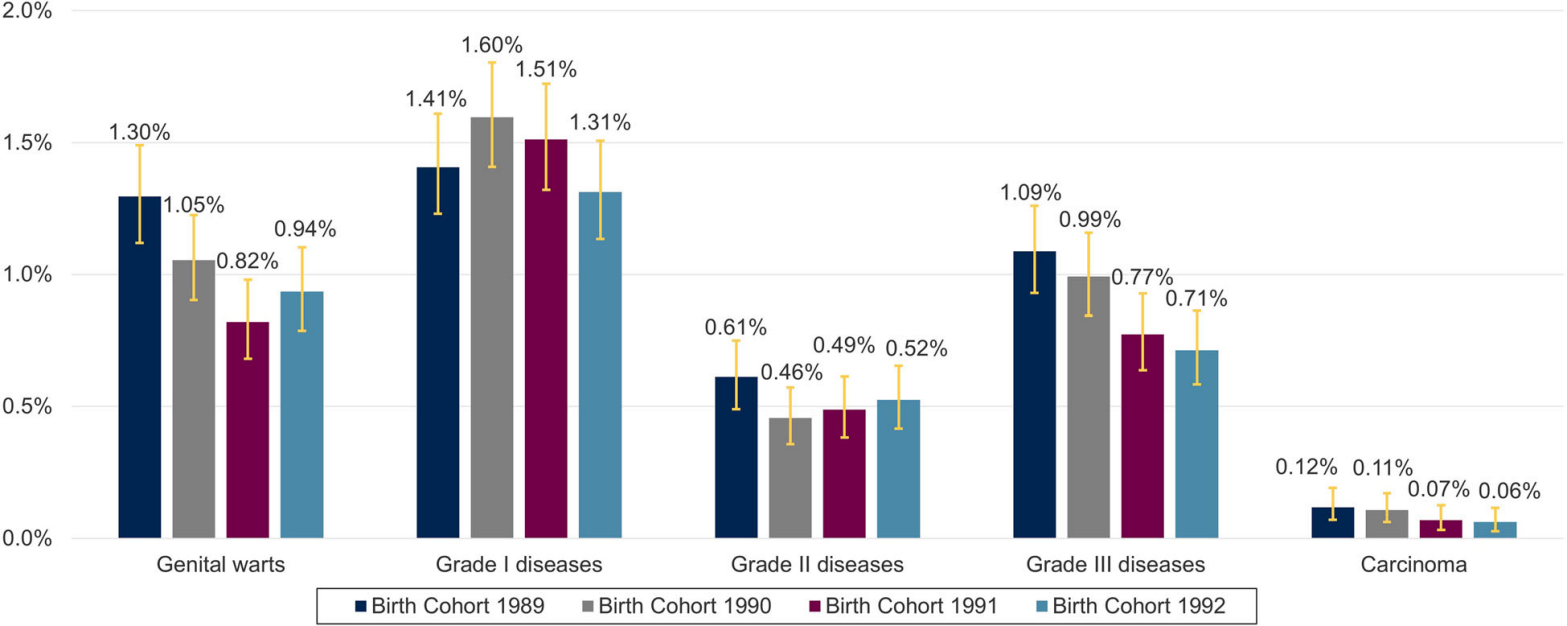
Three-year APR of anogenital diseases in women 23–25 years in Germany from 2012 to 2017. APR = Administrative prevalence rate

The highest 3-year administrative APR in all birth cohorts was observed for anogenital diseases grade I (1.41% in birth cohort 1989; 1.31% in birth cohort 1992), followed by genital warts (1.30% in birth cohort 1989; 0.94% in birth cohort 1992) and anogenital diseases grade III (1.09% in birth cohort 1989, 0.71% in birth cohort 1992). Anogenital diseases grade II (0.61% in birth cohort 1989, 0.52% in birth cohort 1992) and especially invasive carcinoma (0.12% in birth cohort 1989, 0.06% in birth cohort 1992) were less frequently documented (Fig. 2).

### Genital warts

The highest 3-year APR for genital warts was observed for birth cohort 1989 with 1.30% (1.12–1.49). A lower 3-year APR was observed for the younger cohorts (0.94% (0.79–1.10) birth cohort 1992) (all birth cohorts in Fig. 2). The 1-year APR for genital warts is summarized for individual age years in the supplement (see ADDITIONAL FILE 1, Supplementary Figure 1).

### Anogenital diseases grade I

**Fig. 3:**
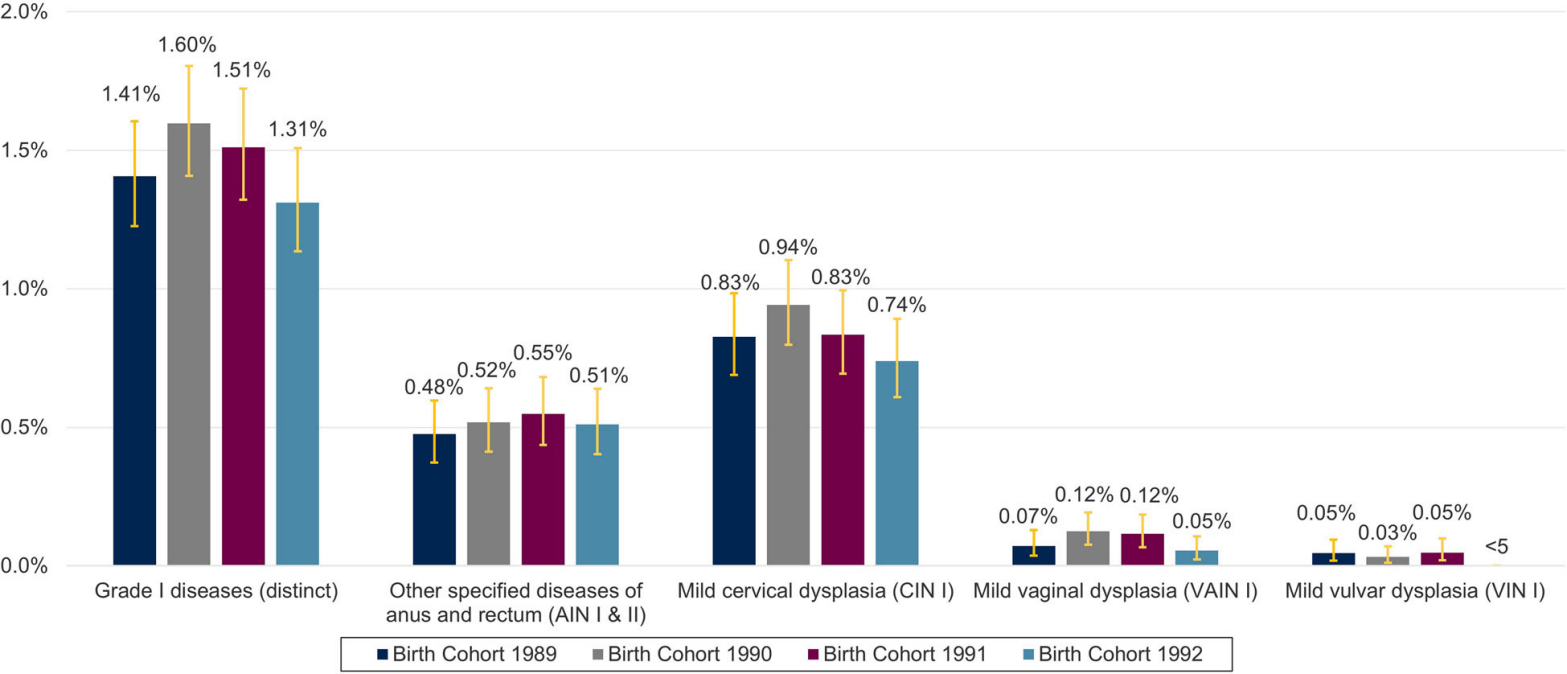
Three-year APR of anogenital diseases grade I in 23–25-year-old women in Germany from 2012 to 2017**.** APR = Administrative prevalence rate; AIN = anal intraepithelial neoplasia; CIN = cervical intraepithelial neoplasia; VAIN = vaginal intraepithelial neoplasia; VIN = vulvar intraepithelial neoplasia. The figure shows the 3-year APR of anogenital diseases grade I (total and the subsets of conditions in grade I category). Please note, that AIN I & II are coded via the same ICD-10-GM code. Due to data protection regulations results for patients with *n* < 5 must not be reported and can constitute 1 to 4 patients

Three-year APR trend for anogenital diseases grade I remained stable (1.41% (1.23–1.61) in birth cohort 1989 to 1.31% (1.14–1.51) in birth cohort 1992). A peak was observed in birth cohort 1990 (1.60% (1.41–1.80). Among the anogenital diseases grade I, mild cervical dysplasia (CIN I) was most frequently recorded, followed by other specified diseases of anus and rectum (AIN I & II). With a 3-year APR of a maximum of 0.12% for mild vaginal dysplasia (VAIN I) and 0.05% for mild vulvar dysplasia (VIN I) these two anogenital diseases were less frequently recorded compared with CIN I and AIN I & II (Fig. 3). 1-year APR for grade I disease are summarized for individual age years in the ADDITIONAL FILE 1, Supplementary Figure 2.

### Anogenital diseases grade II

**Fig. 4:**
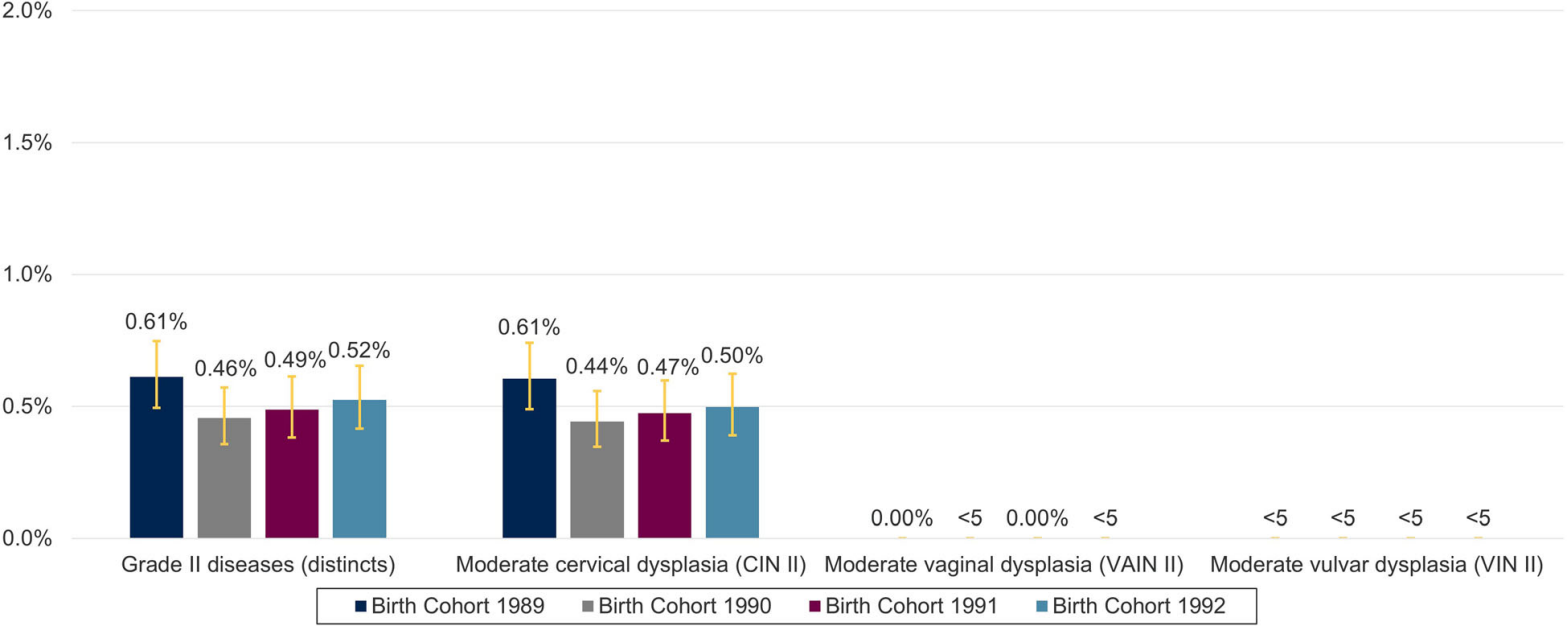
Three-year APR of genital diseases grade II in 23–25-year-old women in Germany from 2012 to 2017. APR = Administrative prevalence rate; CIN = cervical intraepithelial neoplasia; VAIN = vaginal intraepithelial neoplasia; VIN = vulvar intraepithelial neoplasia. The figure shows the 3-year APR of anogenital diseases grade II (total and the subset of conditions in the grade II category). Due to data protection regulations results for patients with *n* < 5 must not be reported and can constitute 1 to 4 patients

Overall, the 3-year APR for anogenital diseases grade II remained stable over the four birth cohorts. It only decreased slightly from 0.61% (0.49–0.75) in birth cohort 1989 to 0.52% (0.42–0.65) in birth cohort 1992. Among the anogenital diseases grade II moderate cervical dysplasia (CIN II) was most frequently recorded. Moderate vaginal dysplasia (VAIN II) and moderate vulvar dysplasia (VIN II) were hardly recorded (Fig. 4). 1-year APR for grade II disease are summarized for individual age years in the ADDITIONAL FILE 1, Supplementary Figure 3.

### Anogenital diseases grade III

**Fig. 5:**
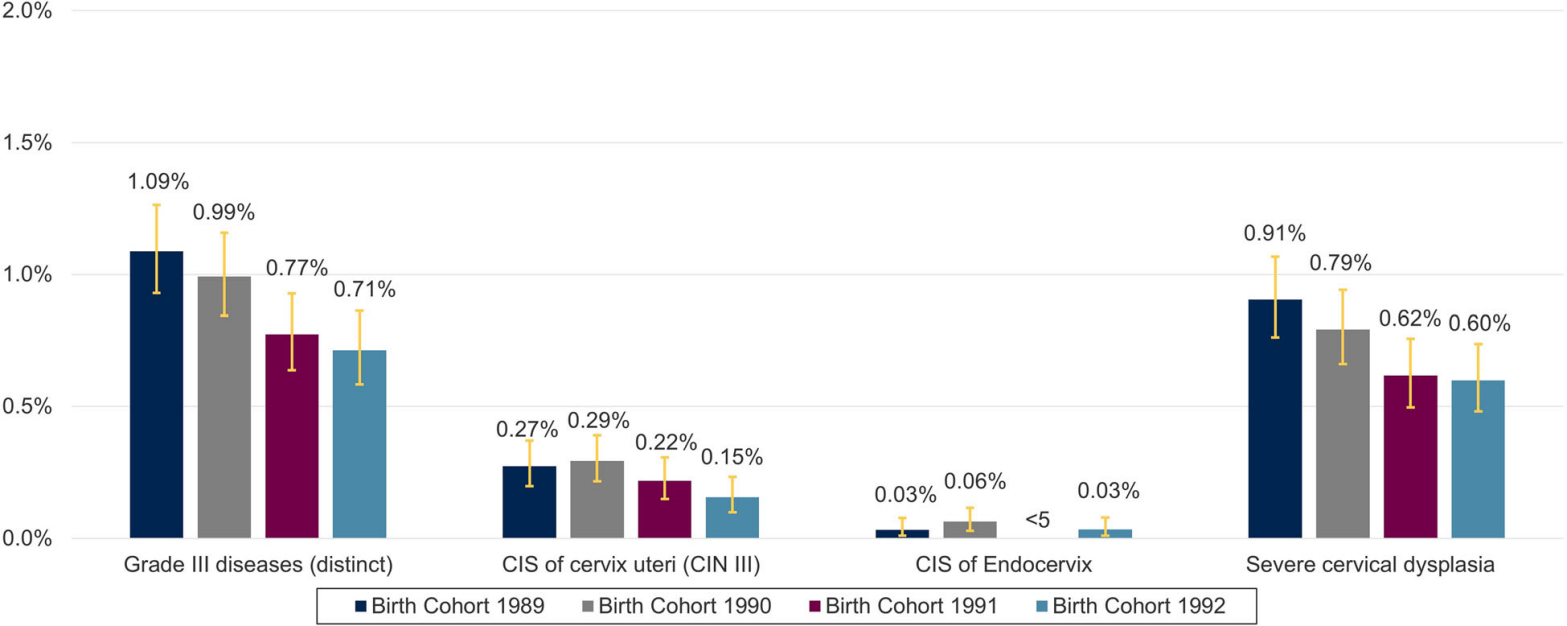
Three-year APR of genital diseases grade III in 23–25-year-old women in Germany from 2012 to 2017. APR = Administrative prevalence rate, CIS = Carcinoma in situ, AIN = anal intraepithelial neoplasia; CIN = cervical intraepithelial neoplasia; VAIN = vaginal intraepithelial neoplasia; VIN = vulvar intraepithelial neoplasia. The figure shows the 3-year APR of anogenital diseases grade III (total and the subset of conditions in the grade III category). AIN III, VIN III, VAIN III, and other severe vaginal and vulvar dysplasia has been excluded from this figure as patient counts were either *n* = 0 or *n* < 5. Due to data protection regulations results for patients with *n* < 5 must not be reported and can constitute 1 to 4 patients

The 3-year APR for anogenital diseases grade III decreased continuously from birth cohort 1989 (1.09% (0.93–1.26)) to birth cohort 1992 (0.71% (0.58–0,86)). Among the anogenital diseases grade III, severe cervical dysplasia was most frequently recorded and followed the trend of the pooled anogenital diseases grade III results. (Fig. 5). 1-year APR for grade III disease are summarized for individual age years in the ADDITIONAL FILE 1, Supplementary Figure 4.

### Invasive carcinoma

The 3-year APR for invasive carcinomas decreased from 0.12 to 0.06% (0.07–0.19 in birth cohort 1989 and 0.03–0.11 in birth cohort 1992) (Fig. 2). Malignant neoplasms of the cervix uteri were most frequently recorded and were responsible for almost all recorded diagnoses for carcinomas in all birth cohorts. Malignant neoplasms of anus and anal canal were not recorded in the analyzed birth cohorts.

## Discussion

The aim of this study was to describe the burden of potentially HPV-related anogenital diseases in 23–25 old women after the introduction of HPV vaccination based on German administrative statutory health insurance claims data for the years 2012 to 2017. The three birth cohorts 1990 to 1992 were included, as they were fully eligible to receive HPV vaccination according to the first STIKO recommendation for HPV in 2007 and further birth cohort 1989, which was partially eligible (girls of birth cohort 1989, who turned 18 years old before March 23rd, 2007 exceeded the recommended age for vaccination in 2007). We found the highest administrative prevalence for anogenital diseases grade I, followed by genital warts and anogenital diseases grade III among all analyzed birth cohorts. A lower burden of anogenital disease potentially related to HPV was observed in the younger birth cohorts as compared to the older cohort. This was observed especially for genital warts and anogenital disease grade III.

The following section discusses the results in the context of HPV vaccination coverage in Germany. HPV vaccination status of the study population could not be evaluated, as the included birth cohort should have received their HPV vaccination in the years 2007 to 2010, which were not available for this analysis. For the analyzed birth cohorts two publications provide estimates on the HPV vaccination coverage. Hense et al. evaluated initiation of HPV vaccination in 2008 based on data from a statutory health insurance and reported 37% of 16 years old girls with at least one dose, which would overlap with birth cohort 1992. In the same publication rates were 33, 19 and 18% for 17-, 18-, and 19-year-old women, respectively (corresponding to birth cohorts 1992–1989) [19]. Delere et al. reported HPV vaccination coverage based on self-reported history of approximately 2000 women aged between 18 and 20 years old in 2010 (cohorts 1990–1992) with 48.5% for three doses and 60.2% for at least one dose [24]. While these figures are to be interpreted with caution as they most likely do not accurately represent the vaccination coverage of our study population in the analyzed time period, it might give three important estimates: 1) coverage rates for HPV vaccination in Germany were generally lower than in countries with vaccination programs in schools [21]. In Australia e.g., the three-dose vaccination coverage for girls turning 15 years of age was 79% in 2015 [25]. Hence, disease burden in birth cohorts eligible for HPV vaccination may still be higher than one might expect with a better vaccination coverage. 2) vaccination coverage increases from birth cohorts that were not entirely eligible for HPV vaccination according to STIKO to birth cohorts that were eligible. Since we found a downward trend in the 3-year APR for selected diagnoses in favor of the younger birth cohorts, it may be speculated that this is due to increasing HPV vaccine coverage in younger cohorts. We found the downward trend in the 3-year APR for genital warts and grade III dysplasia, but not for grade I and II dysplasia. While this might be due to the rather low HPV vaccination coverage in Germany, it might be also correlated with the HPV types that are covered by the vaccines. HPV types causing the majority of genital warts (approximately 90% caused by HPV 6/11) and a number of grade III cervical diseases (approximately 45% caused by HPV 16/18) were covered by HPV vaccines available during the observation period. Grade I disease, however, is caused to a lesser extent by types 6, 11, 16 and 18 [9]. It may be speculated that a vaccine effect might be masked in an analysis without selection for vaccine-HPV type-associated disease. And 3), also for birth cohort 1989, which was not entirely eligible for HPV vaccination according to STIKO recommendation, a coverage of approximately 18% was reported in 2008 [19]. This birth cohort turned 18 during the year 2007 and thus girls may have had the chance to receive the vaccination regularly before their birthday. Also, individual health insurances provided an extended reimbursement for HPV vaccination up to the age of 26, or the vaccine may have been purchased as self-payer. Hence, in none of the analyzed birth cohorts our prevalence rates for HPV diseases do reflect the anogenital disease burden in a population without HPV immunization. Therefore, no assumptions on burden of potentially HPV-related anogenital diseases in unvaccinated women between 23 and 25 years of age can be made.

This next section discusses the results in the context of previous studies on burden of HPV diseases in Germany. One previous German study examined HPV type prevalence [24] but has not investigated associated anogenital diseases. Further studies assessing the incidence of anogenital warts before and after the introduction of the HPV vaccination in German statutory health insurance members aged 10–79 years old reported the highest incidence of anogenital warts for the age group 20–24 years [4, 6, 26]. In 2010, incidence was 493 per 100,000 person years in 20–24 year old and 149 per 100,000 person years in 15–19 year old females [6]. In a population-based surveillance study conducted from 2009 to 2010 in Wolfsburg/Germany to investigate the burden of HPV infections and associated anogenital diseases in women who were born in 1988 or 1989 (and 1983/84), an incidence of 0.72% and a total life risk of 1.4% for genital warts was estimated for the cohort 1988/89 [27]. For the same cohort a prevalence of 0.83% for CIN II and 0.33% for CIN III diseases was detected [28]. The 3-year APR for anogenital warts of 1.30% and for grade III cervical dysplasia of 0.60% for birth cohort 1989 found in our study is generally in line with this dimension. In Australia an observational study based on clinical diagnoses reported a prevalence of genital warts of 18.4% in young women before the introduction of HPV vaccination [29]. On one hand, the remarkably lower burden in our study might be explained by the fact that our analyzes did not include a population in the pre-HPV-vaccine era, and on the other hand the use of administrative claims data in our study may have resulted in an underestimation of diagnoses (see further discussion on limitations below). It is also important to note that our analysis only provides a snapshot of the APR of anogenital diseases in women of a selected, young age (23–25) and are not transferable to the prevalence rates of HPV-related anogenital diseases over the complete lifespan.

The following section discusses the results more specifically in the context of previous studies on HPV vaccination impact in Germany. Single previous reports focused on HPV vaccine impact in Germany and are in line with our results. Deleré et al. found a significant lower HPV 16/18 prevalence in vaccinated women suggesting first effects of the vaccination. Data from the German Pharmacoepidemiological Research Database (GePaRD) also demonstrated a decline of anogenital warts among males and females at the age of 14–24 comparing the timeframe prior to vaccination with the timeframe after vaccination. While the largest decrease (by 60%) was observed for women in the age group 16–20, the incidence ratio in women aged between 21 and 26 years was reduced by 10–20% [6]. In our study, genital warts have been reduced by 28% (from 1.30% in birth cohort 1989 to 0.94% in birth cohort 1992), and HPV related anogenital diseases grade III have been reduced by 35% (from 1.09% in birth cohort 1989 to 0.71 in birth cohort 1992).

This last section discusses further potential limitations of the study in detail, most inherent with the use of health insurance claims data. Claims data are primarily collected for reimbursement purposes. Therefore, only patients who seek physician treatment and cause reimbursement for the health insurance could be identified in the database. Patients without symptoms might not seek medical advice, individuals who do not participate in screening examinations might not be identified, and patients who treat their disease (e.g. genital warts) by themselves or ignore the condition are not recorded in the database. Therefore, this study only presents the administrative prevalence based on reimbursement data and not the clinical prevalence of potentially HPV-related anogenital diseases on a population level. Furthermore, Germany had an opportunistic cervical cancer screening beginning at the age of 20 and thus, most documented diagnoses of intraepithelial neoplasia most likely have been identified during screening and further work-up (cytologically or histologically). The annual and biannual cervical cancer screening participation rate in women 25–29 years old was reported with approximately 60 and 70% in 2011, respectively [30]. As not all women attend the screening program for cervical cancer it is likely that some of the diseases might not have been diagnosed and recorded by a physician. Therefore, our results might underestimate the true clinical prevalence of the assessed anogenital diseases in Germany for women between 23 and 25 years.

For the identification of potentially HPV related anogenital diseases we used ICD-10-GM codes, which is the official classification for the encoding of diagnoses in inpatient and outpatient medical care in Germany since 2000 [31]. Clinicians in the outpatient setting are required to add one of the following specifications to the ICD-10-GM codes: “suspected diagnosis”, “diagnosis ruled out”, “status post”, or “verified diagnosis”. For instance, “suspected” may be coded, if the physician is not certain about the presence of the coded disease and a confirming laboratory analysis is still pending. To ensure the accuracy of diagnoses only “verified” diagnoses in the outpatient and primary and secondary diagnoses in the inpatient sector were used. With this approach however, we excluded women who might be suspected to have one of the investigated anogenital diseases or women who have been cured from of the anogenital diseases (e.g. genital warts) and see their physician for a follow-up visit. This could have led to an underestimation of the APR. Additionally, we decided to only consider very specific ICD-10-GM codes potentially associated with HPV-related anogenital diseases, but physicians may use less specific codes. This might also have contributed to the underestimation of the true rate of clinical diagnoses.

Cervical cancer screening in Germany is Pap-based for women in their twenties. Documented diagnoses in our analysis are most likely cytologically or histologically defined. No laboratory data was available for this study to demonstrate an HPV association of anogenital diseases. Therefore, it is not possible to distinguish between anogenital diseases, which were caused by an HPV infection and those not caused by an HPV infection. However, it is expected that high-risk HPV infections cause almost all cervical cancers and precancers, approximately 90% of high-grade anal, vulvar and vaginal intraepithelial neoplasia and 30, 70 and 90% of vulvar, vaginal and anal cancers, respectively [9] and therefore, a very high rate of HPV association is expected for our captured diagnoses.

Finally, the instrument of ARP is subject to limitations that might influence the results of our study. Data on outcomes might be collected in different ways over time for the different study cohorts. Migration of populations affecting the study cohort might influence the differences between the groups. Seasonal or cyclical variations might result in fluctuations that affect the outcome trend. These limitations are assumed to be negligible in our study as we expect no major changes in the recording behavior of physicians during the study period and the impact of migration will not have affected the SHI, as medical services are paid by other payers [32]. Seasonal or cyclical variations in HPV types have not been reported in the literature so far.

## Conclusions

In summary, our results demonstrate the highest administrative prevalence for anogenital diseases grade I, followed by genital warts and anogenital diseases grade III among all analyzed birth cohorts. Even though the HPV vaccination status of the study population was unknown, a decrease of the disease burden of genital warts and anogenital grade III disease was observed in favor of the younger birth cohorts who were fully eligible for HPV vaccination according to STIKO recommendation. Further research is necessary to confirm the observed trend including analyses linked to vaccination status.

## Supplementary information

**Additional file 1.**

## Data Availability

The utilized database in this study is available from the Institute for Applied Health Research Berlin (InGef) but restrictions apply to the availability of these data. Access to the data is restricted to health service research and is granted on a study by study basis from the InGef on behalf of the participating statutory health insurances.

## Abbreviations

AIN: Anal intraepithelial neoplasia
APR: Administrative prevalence rate
CIN: Cervical intraepithelial neoplasia
CIS: Carcinoma in situ
EMA: European Medicines Agency
GePaRD: German Pharmacoepidemiological Research Database
HPV: Human papillomavirus
ICD-10-GM: International Statistical Classification of Diseases, German Modification
InGef: Institute for Applied Health Research Berlin [Institut für Angewandte Gesundheitsforschung Berlin]
OTC: Over-the-counter
SHI: Statutory health insurance [Gesetzliche Krankenversicherung]
STIKO: German Standing Committee on Vaccination [Ständige Impfkommission]
VAIN: Vaginal intraepithelial neoplasia
VIN: Vulvar intraepithelial neoplasia
ZfKD: German Centre for Cancer Registry Data [Zentrum für Krebsregister Daten]
