# Supplementary material for: Burden of HPV related anogenital diseases in young women in Germany – an analysis of German statutory health insurance claims data from 2012 to 2017
Source: BMC Infect Dis. 2020 Apr 22;20:297. doi: 10.1186/s12879-020-05002-w (PMC7178589; doi:10.1186/s12879-020-05002-w)
Supplement: Supplementary file 1 — Additional file 1. [file 12879_2020_5002_MOESM1_ESM.docx]

# Additional File 1

***1-Year Administrative Prevalence***

***Genital warts***

Younger birth cohorts had a lower 1-year APR compared with birth cohort 1989, except for birth cohort 1992. The 1-year APR in birth cohort 1989 was increasing constantly from age 23 (0.48% (0.37-0.60)) to 25 (0.62% (0.50-0.76)) years. While birth cohorts 1990 and 1991 showed a decrease of the 1-year APR from 23 to 25 years (from 0.49% (0.39-0.61) to 41% (0.32-0.52) in birth cohort 1990 and from 0.39% (0.30-0.51) to 0.30% (0.22-0.40) in birth cohort 1991), birth cohort 1992 (0.36% (0.27-0.47) to 0.42% (0.33-0.54)) showed an increase of the 1-year APR from 23-25 years as seen for birth cohort 1989 (Supplementary Figure 1).

**Supplementary Figure 1** One-year administrative prevalence of genitals warts in women 23-25 years in Germany from 2012-2017

***Anogenital disease grade I***

Looking at single age years, the 1-year APR in birth cohort 1989 showed the highest increase from 23 (0.46% (0.36-0.58)) to 25 (0.72% (0.60-0,87)) years. While the 1-year APR in birth cohort 1990 increased slightly from 23 (0.64% (0.52-0.77)) to 25 (0.69% (0.56-0.83)) years, birth cohort 1991 indicated a decrease of 1-year APR from 23 (0.68% (0.56-0.83)) to 25 (0.56% (0.45-0.70)) years. The 1-year APR in birth cohort 1992 remained almost constantly (0.52% (0.41-0.65) at the age of 23 and 0.55% (0.43-0.68) at the age of 25) (Supplementary Figure 2).

**Supplementary Figure 2** One-year administrative prevalence of anogenital diseases grade I in women 23-25 years in Germany from 2012-2017

***Anogenital disease grade II***

Analyzing the trend at single age years, the 1-year APR in all analyzed birth cohorts indicated an increase from 23 to 25 years. The highest increase of the 1-year APR was observed in birth cohort 1990 from 23 (0.12% (0.08-0.19)) to 25 (0.26% (0.19-0.35)) years, followed by birth cohort 1989 and 1991 from 23 (0.18% (0.12-0.26) in birth cohort 1989 and 0.16% (0.10-0.24) in birth cohort 1991) to 25 (0.31% (0.23-0.41) in birth cohort 1989 and 0.22% (0.15-0.31) in birth cohort 1991). Although the 1-year APR in birth cohort 1992 decreased slightly by 0.02 percentage points from 23 to 24 years, overall, it increased from 23 (0.22% (0.15-0.31)) to 25 (0.25% (0.18-0.34)) years (Supplementary Figure 3).

**Supplementary Figure 3** One-year administrative prevalence of anogenital diseases grade II in women 23-25 years in Germany from 2012-2017

***Anogenital disease grade III***

Looking at single age years, birth cohort 1989 showed the highest increase of 1-year APR from 23 (0.25% (0.18-0.34)) to 25 (0.64% (0.52-0.78)) years, followed by birth cohort 1990 from 23 (0.39% (0.30-0.50)) to 25 (0.48% (0.38-0.60)) years. Although the 1-year APR in birth cohort 1990 and 1991 decreased by 0.10 percentage points (birth cohort 1991) and 0.05 percentage points (birth cohort 1991) from 23 to 24 years, they remained constantly from 23 (0.37% (0.28-0.48) in birth cohort 1991 and 0.33% (0.24-0.44) in birth cohort 1992) to 25 (0.38% (0.29-0.49) in birth cohort 1991 and 0.32% (0.24-0.43%) in birth cohort 1992) years (Supplementary Figure 4).

**Supplementary Figure 4** One-year administrative prevalence of anogenital diseases grade III in women 23-25 years in Germany from 2012-2017
